# Supplementary material for: Transcriptomics and RNA-Based Therapeutics as Potential Approaches to Manage SARS-CoV-2 Infection
Source: Int J Mol Sci. 2022 Sep 21;23(19):11058. doi: 10.3390/ijms231911058 (PMC9570475; doi:10.3390/ijms231911058)
Supplement: Supplementary file 1 [file ijms-23-11058-s001.zip › ijms-1871678-supplementary.pdf]

Supplementary Information.

Table S1. Comparison of the most commonly used sequencing platforms for SARS-CoV-2 sequencing.

**Platform**	**Cost and maintenance**	**Advantages**	**Limitations**	**Genome SARS-CoV-2 cost and coverage**	**Sequencing yield and samples per run**	**Pipeline**
Illumina	Moderate sequencing cost, high start-up cost. Frequent maintenance [240].	High capacity to sequence large volumes of samples. High accuracy in SNV. Well-established methods of analysis [240]. Clinical application use [241]	High cost of maintenance. 300pb maximum read size [240].	32.5 USD per genome on Illumina NextSeq 550. Coverage 300-600x, ≥ 99%, [242,243] Sample viral load up to 30 Ct value	1.2-6000Gb[243] 12-3072 samples per run.	Base calling and demultiplexing with Bcl2Fastq [244]. Removal adaptors and trimming with Cutadapt [245] and Trimmomatic [246]. Alignment with Bowtie2 [247]. Variant calling with Samtools mpileup and BCFtools [248].

Oxford Nanopore	Low cost of sequencing, low start-up cost. Without maintenance [240].	Very portable sequencer. It is obtaining data in real-time. Long reads 13-20 kb. RNA-direct sequencing [240].	They are constantly changing analysis methods. Moderate accuracy in SNV [249]. Homopolymers complicated to sequence.	29 USD per genome on MinION[249]. Coverage 800-1000x, ≥ 99%, Sample viral load up to 31 Ct value [249]	1.8-245Gb[250] 12-2,304 samples per run.	Base calling and demultiplexing with Guppy [251]. Alignment with Minimap2 [252]. Variant calling with Nanopolish [253] or Medaka [254].
Ion Torrent	Moderate start-up and sequencing cost. Frequent maintenance [240].	Library generation automatic. High accuracy in SNV. Fast sequencing.	Homopolymers complicated to sequence.	122 USD per genome on GeneStudio S5 Coverage 3,000x, ≥ 99%, [255] Sample viral load up to 38 Ct value[256]	0.5-50Gb[257] 2-80 samples per run.	Similar pipeline to Illumina can be used.
Pacific Biosciences	Moderately high cost of sequencing and start-up. Frequent maintenance [240].	Long reads 10-16 kb. Sequencing genomic regions such as high/low G + C, tandem repeat, and interspersed repeat regions. RNA-direct	High raw error rate up to 8%, Low accuracy in SNV [258].		18-680Gb [259] Up to 900 samples per run [260].	Demultiplexing with lima, USEARCH to reoriented 5’-3’ direction, Cutadapt for trimming. Variant calling with Minimap2 [252].

sequencing [240].
